# Systemic Embolism From Aortic Arch Thrombus Following Warfarin Discontinuation: A Case Report

**DOI:** 10.7759/cureus.84319

**Published:** 2025-05-18

**Authors:** Abdelrahman N Elrefaeei, Omar F Al-Nahhas, Mohammed F Al-Nahhas, Thiagarajan Jaiganesh

**Affiliations:** 1 Emergency Medicine, Tawam Hospital, Al Ain, ARE; 2 Clinical Sciences, Ajman University, Ajman, ARE; 3 General Surgery, RAK College of Medical Sciences, Ras Al Khaimah, ARE

**Keywords:** anticoagulation discontinuation, basilar artery thrombosis, cerebral embolism, embolic stroke, enoxaparin, mechanical thrombectomy, mural thrombus, upper limb ischemia, warfarin cessation

## Abstract

We present the case of a 40-year-old female with a previous diagnosis of unprovoked deep vein thrombosis (DVT), who had been on long-term warfarin therapy but discontinued it one month before presentation. She arrived at the emergency department following two episodes of transient loss of consciousness at work, raising concerns for a possible cerebrovascular event. Further evaluation revealed evidence of systemic embolization, as she developed acute basilar artery thrombosis and left upper limb ischemia findings consistent with multiple embolic events. Computed tomography angiography (CTA) demonstrated a mural thrombus in the thoracic aorta and occlusion of the left brachial artery. The patient received intravenous recombinant tissue plasminogen activator (rTPA), followed by successful mechanical thrombectomy of the basilar artery. She was managed with systemic anticoagulation and discharged neurologically intact. This case highlights the critical importance of maintaining anticoagulation in high-risk individuals and demonstrates the potentially life-threatening consequences of abrupt discontinuation.

## Introduction

Basilar artery thrombosis is a rare but potentially fatal form of posterior circulation stroke, accounting for approximately 1%-4% of all ischemic strokes, and is considered one of the most challenging cerebrovascular emergencies to diagnose and manage [1-2]. Simultaneously, peripheral arterial embolism, particularly involving the upper limb, is uncommon but clinically significant, with an estimated incidence of 0.9-2% among all embolic events [3]. The presence of both basilar artery thrombosis and upper limb peripheral arterial embolism in a single patient raises strong suspicion for a central embolic source, especially in individuals with a recent history of anticoagulation discontinuation [4]. One such source is thoracic aortic mural thrombus (TAMT), which can develop on apparently normal aortic walls without aneurysm or dissection, making it diagnostically elusive. TAMT poses a significant clinical risk as it can fragment and embolize distally, causing ischemic events in multiple vascular territories [5]. This case emphasizes the clinical implications of abruptly stopping oral anticoagulation (OAC) therapy in patients with a history of venous thromboembolism (VTE). This case emphasizes the clinical implications of abruptly stopping oral anticoagulation (OAC) therapy in patients with a history of VTE. 

## Case presentation

A 40-year-old female with a past medical history of unprovoked DVT, previously managed with an unknown warfarin dosage for seven years, presented to the emergency department (ED) following two episodes of transient loss of consciousness at work. She had discontinued warfarin one month prior without medical advice.

On arrival, her Glasgow Coma Scale (GCS) score was 15/15. While in the ED, she experienced a sudden, transient decline in consciousness with a GCS of 5/15, followed by spontaneous recovery. Vital signs showed a significant discrepancy in upper limb blood pressures, with a right arm blood pressure of 150/80 mmHg and a left arm blood pressure of 110/70 mmHg. The left radial pulse was absent, raising concern for arterial compromise.

**Figure 1:**
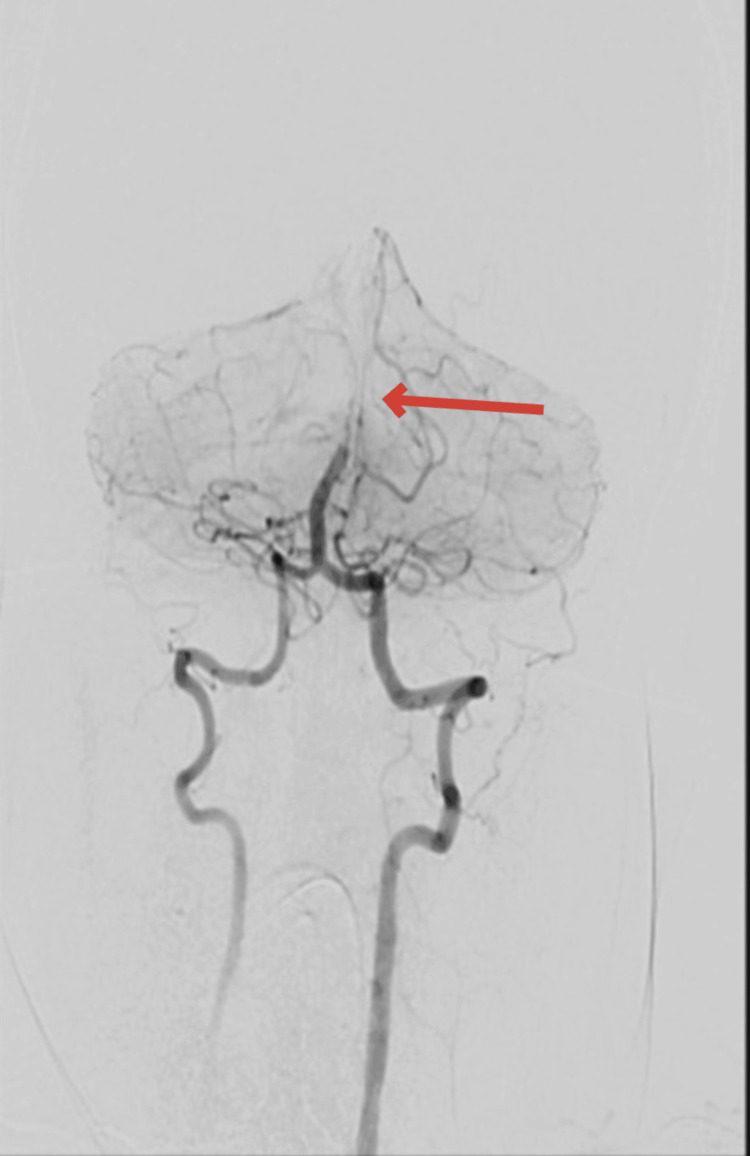
Cervico-cerebral angiogram showing a focal filling defect at the tip of the basilar artery, measuring approximately 4 mm.

**Figure 2:**
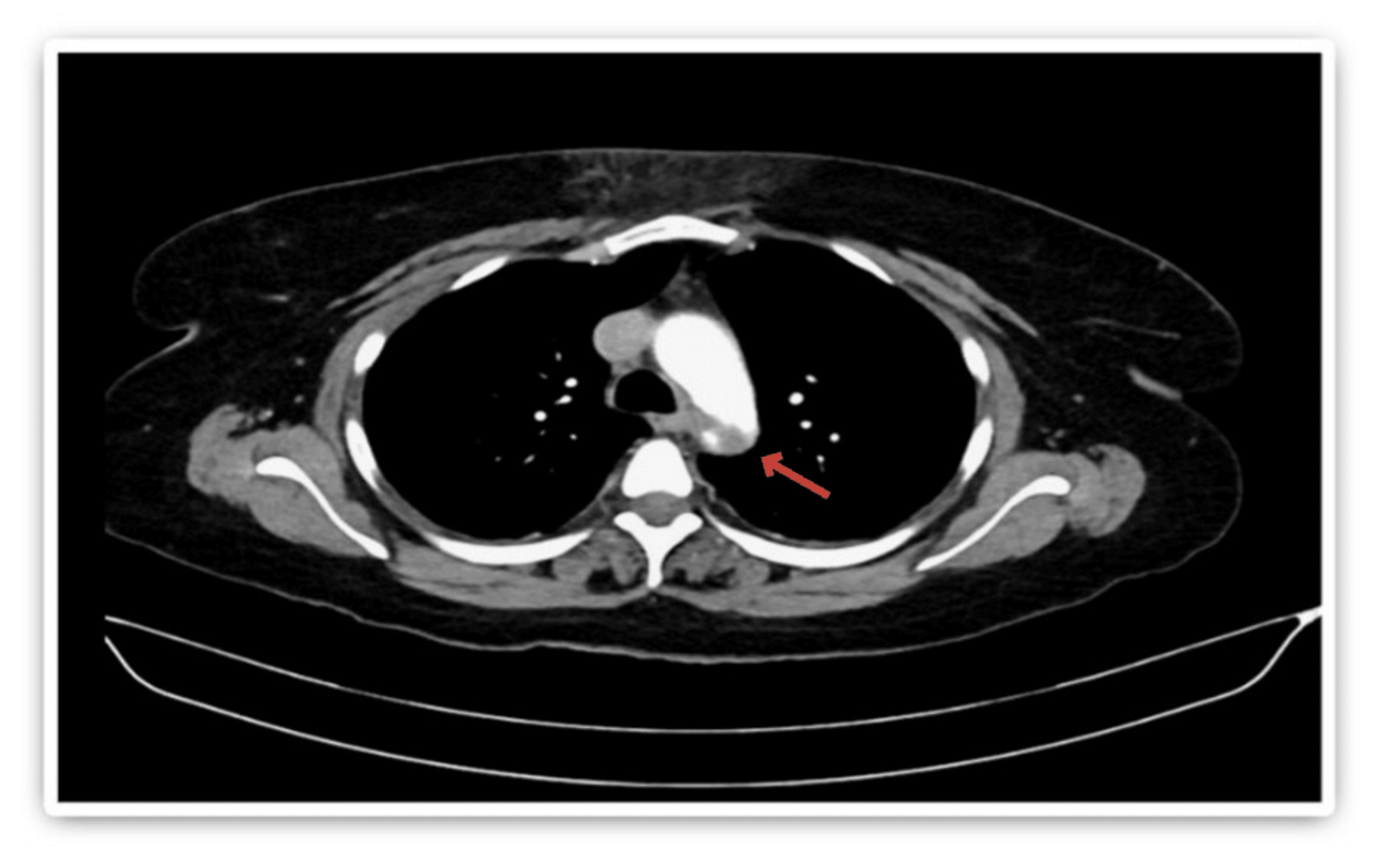
Axial section of CT chest showing an eccentric mural thrombus along the superior aspect of the distal thoracic aortic arch.

**Figure 3:**
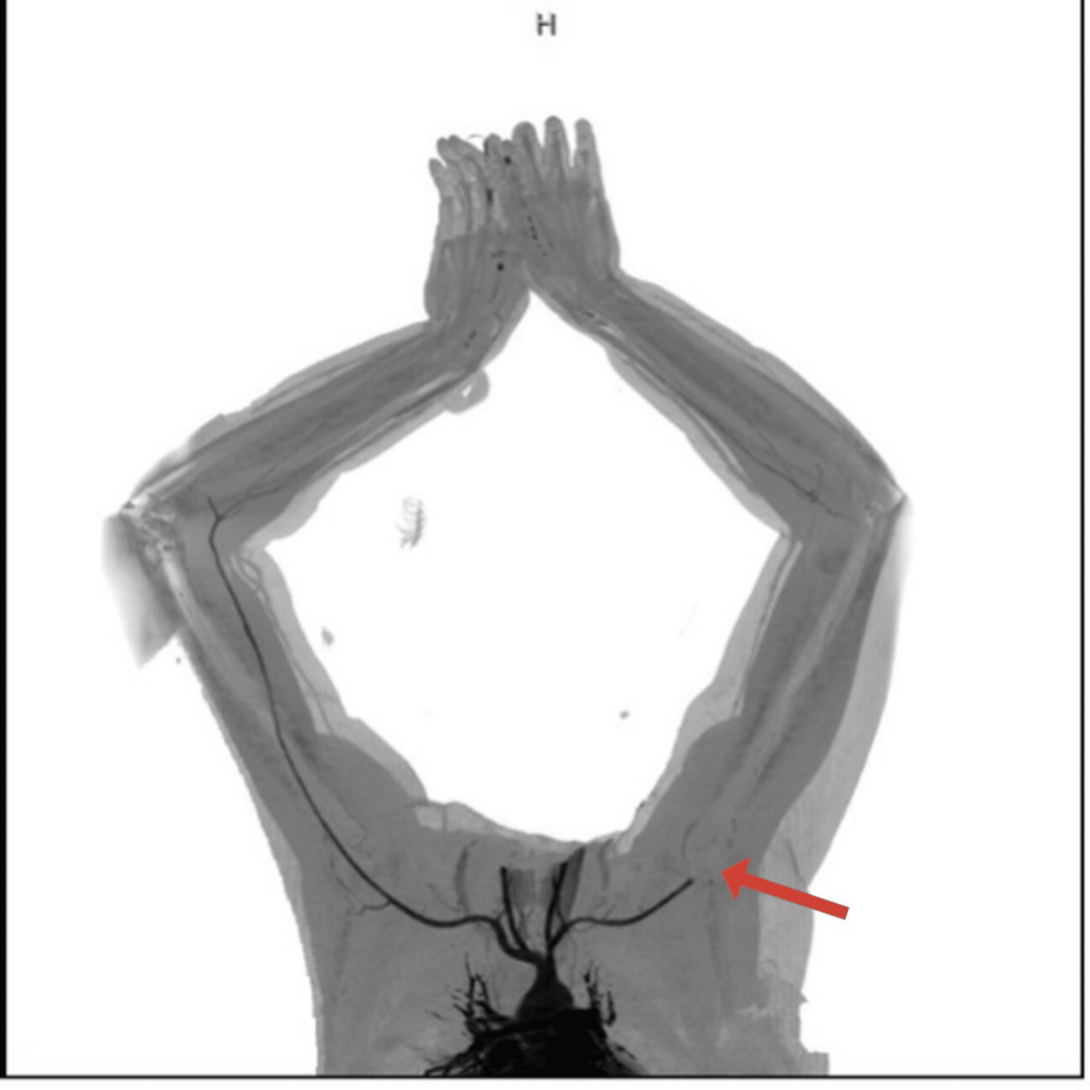
CT angiography of the upper limbs showing complete occlusion of the left brachial artery, likely of embolic origin.

Stroke protocol was activated, and initial laboratory investigations included a complete blood count with differential, urea and electrolytes, coagulation panel, blood type and crossmatch, serum beta-human chorionic gonadotropin (beta-hCG), venous blood gas (VBG), and urine and blood toxicology screening. All initial results were unremarkable. Coagulation studies showed an International Normalized Ratio (INR) of 1.04, Prothrombin Time (PT) of 11.4 seconds, Activated Partial Thromboplastin Time (APTT) of 24.6 seconds, and a markedly elevated D-dimer of 28.500 mg/L. Non-contrast CT brain was unremarkable. Subsequent computed tomography angiography (CTA) of the head, neck, and aorta revealed a focal filling defect (~4 mm) in the mid-basilar artery (Figure 1). A mural thrombus in the proximal descending thoracic aorta, distal to the left subclavian artery, measuring 13 mm in depth and 22 mm in anteroposterior dimension (Figure 2). Complete occlusion of the left brachial artery at the elbow (Figure 3).

**Figure 4:**
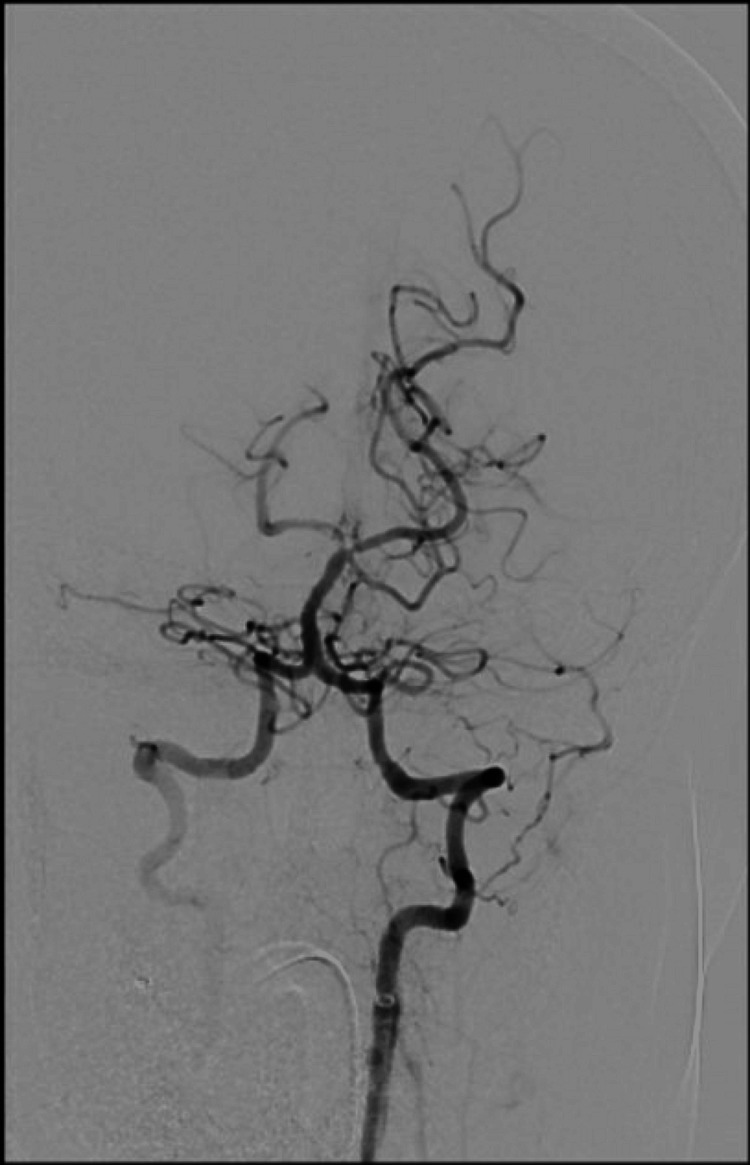
Post-thrombectomy cervico-cerebral angiogram. Recanalization of the basilar artery post-thrombectomy.

The patient received intravenous recombinant tissue plasminogen activator (rTPA) and underwent emergency mechanical thrombectomy, which resulted in successful recanalization of the basilar artery. Post-procedure imaging revealed a non-flow-limiting filling defect in the right posterior cerebral artery (Figure 4).

She was admitted to the intensive care unit (ICU), where she was intubated, ventilated, and started on intravenous unfractionated heparin. Norepinephrine was required for transient hemodynamic support. Remarkably, she self-extubated the following day with a GCS of 14/15. Peripheral Doppler confirmed reperfusion of the left upper limb.

Magnetic resonance imaging (MRI) of the brain showed no evidence of infarction. Repeat CTA of the head and neck on day 2 demonstrated patency of the posterior circulation. The patient was transitioned to therapeutic enoxaparin 100 mg subcutaneously twice daily and discharged with no neurological deficits.

**Figure 5:**
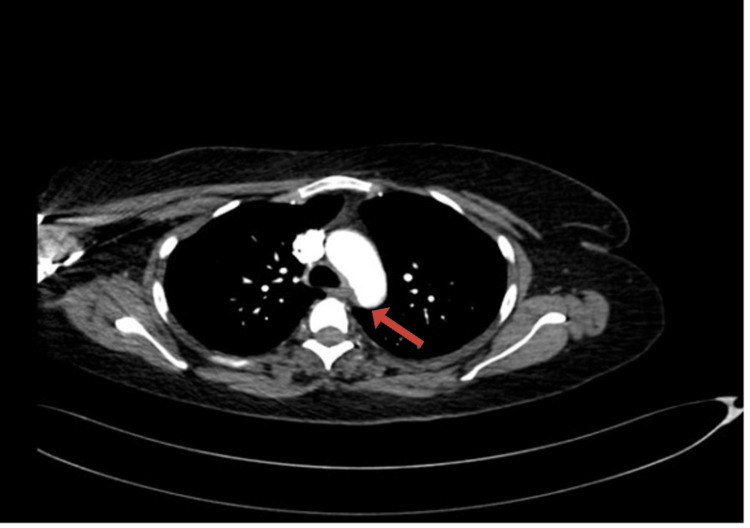
Axial CT chest after 42 days of anticoagulation showing a reduction in the size of the eccentric mural thrombus.

At six-week follow-up, CTA demonstrated a significant reduction in the size of the thoracic aortic thrombus, now measuring 6 mm (Figure 5).

## Discussion

This case underscores the severe and potentially life-threatening consequences of abrupt discontinuation of OAC therapy in patients with a history of thromboembolic disease. The patient, who had previously been treated for an unprovoked deep vein thrombosis (DVT), ceased warfarin therapy one month before presentation without medical guidance. Her clinical course highlights the heightened risk of thromboembolic events in the immediate period following cessation of anticoagulation. Literature indicates that even short-term interruption of OAC, such as for peri-procedural management, is associated with a 1% risk of thromboembolic events, including ischemic stroke [6]. Retrospective reviews have found that approximately 2.6% of all ischemic strokes are preceded by recent OAC discontinuation [7-9]. Moreover, evidence from patients with atrial fibrillation demonstrates that discontinuation of OAC therapy for as little as seven consecutive days significantly increases the risk of death, stroke/systemic embolism, and myocardial infarction, underscoring the importance of continuous anticoagulation in high-risk patients [5]. This case provides a striking real-world illustration of this association, emphasizing the importance of clinician-led risk stratification and careful planning when considering temporary or permanent interruption of anticoagulation.

Another key element of this case is the presence of an eccentric mural thrombus in the thoracic aorta, which served as a probable embolic source for both the basilar artery occlusion and the peripheral limb ischemia [10]. Although relatively uncommon, thoracic aortic mural thrombi have been increasingly recognized as a cause of systemic embolism, particularly in patients with underlying prothrombotic conditions, including inherited mutations (e.g., Factor V Leiden) and acquired states (e.g., antiphospholipid syndrome, cancer), increase risk of thrombosis or inadequate anticoagulation [11]. In this patient, the thrombus measured 13 mm in thickness and was located just distal to the origin of the left subclavian artery anatomic location associated with a high risk for distal embolization to both cerebral and upper extremity vessels.

Prompt and coordinated multidisciplinary management was essential to the favorable outcome in this case. The administration of intravenous rTPA followed by mechanical thrombectomy resulted in successful recanalization of the basilar artery, with preservation of posterior cerebral circulation. Post-procedural anticoagulation was carefully managed with unfractionated heparin during the acute phase and later transitioned to therapeutic low molecular weight heparin (enoxaparin). The patient’s neurologic function fully recovered, and follow-up imaging confirmed both cerebral vascular patency and significant regression of the aortic thrombus.

This case also draws attention to the diagnostic value of comprehensive vascular imaging in patients with embolic phenomena affecting multiple territories. The identification of the mural thrombus was pivotal in guiding the long-term management plan, including extended anticoagulation and vascular follow-up. Ultimately, this case reinforces the need for adherence to anticoagulation therapy in high-risk individuals, as well as the importance of patient education, vigilant follow-up, and interdisciplinary collaboration in the management of complex thromboembolic presentations.

## Conclusions

This case illustrates the profound risks associated with discontinuing OAC in high-risk individuals. Multiple embolic events, including both cerebral and peripheral arterial occlusions, can arise from a central source such as aortic mural thrombi. Vigilance in anticoagulation management, patient education, and individualized risk assessment before therapy withdrawal is crucial to prevent life-threatening thromboembolic complications.
